# Revelation of core promoter elements and bidirectional regulation of Geminivirus genes in *Escherichia coli*

**DOI:** 10.1016/j.virusres.2026.199697

**Published:** 2026-02-02

**Authors:** Hsiao-En Lin, Ying-Wen Huang, Yi-Chin Lai, Na-Sheng Lin, Yau-Heiu Hsu, Chung-Chi Hu

**Affiliations:** a Graduate Institute of Biotechnology, National Chung Hsing University, Taichung 40227, Taiwan; b Advanced Plant Biotechnology Center, National Chung Hsing University, Taichung 40227, Taiwan

**Keywords:** Intergenic region (IR), Transcription, Transcription regulation, Prokaryotic systems, Cross-kingdom, Geminiviruses

## Abstract

Several geminiviruses have been show to possess cross-kingdom gene expression capability. In addition, the intergenic region (IR) of geminivirus genomes is known to regulate the transcription of viral early genes (complementary-sense, C-sense) and late genes (virion-sense, V-sense) located in opposite directions in viral genomic DNAs. However, the underlying mechanism remained elusive. In this study, the transcriptional regulation activities in the IR of ageratum yellow vein virus isolate NT (AYVV-NT), a monopartite geminivirus, were characterized in *Escherichia coli*, by using a promoter-trapping system. A functional bidirectional promoter core and regulatory elements for C- and V-sense genes were identified in the IR of AYVV-NT. Quantitative analyses revealed differences in promoter activity in various *E. coli* strains, suggesting that promoter regulation is strain-dependent and influenced by bacterial transcription factors. These findings provide insights into how plant-infecting geminiviruses may regulate gene expression in prokaryotic environments and highlight the potential applications of such viral regulatory elements in bacterial expression systems.

## Highlights

• The cross-kingdom gene expression ability of a geminivirus is studied in *Escherichia coli*.
• Effects of different *E. coli* strains on promoter activities were revealed.
• A 36-bp bidirectional promoter core was identified in the IR of AYVV-NT.
• *Cis*-acting regulatory elements were identified for C- and V-sense coding regions.
• Our results elucidated how geminiviruses may regulate gene expression in bacteria.

## Graphical abstract

**Figure:**
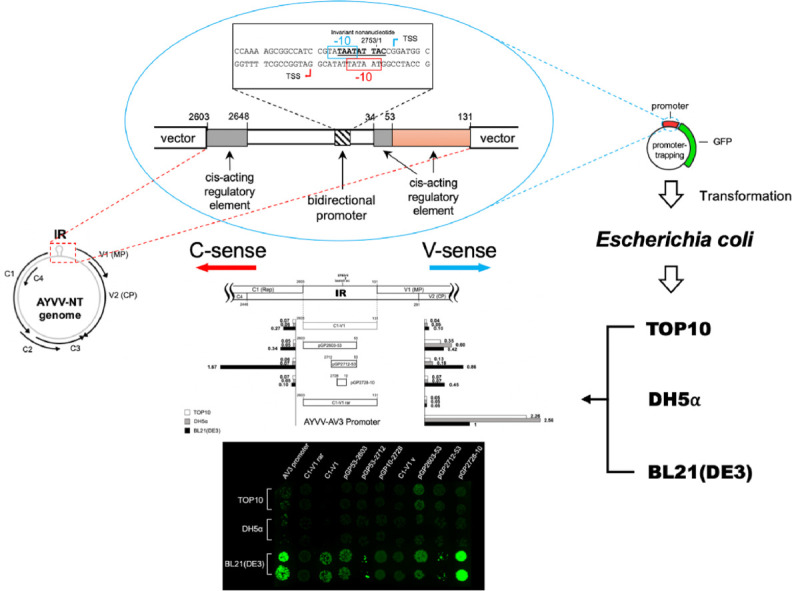

## 1 Introduction

Members of the family *Geminiviridae* are characterized by the geminate-shaped virion structures that encapsidate single-stranded circular DNA (sscDNA) genomes, encoding genes in an ambisense manner. Geminiviruses are pathogens to many important crops, posing serious threats to agricultural production systems worldwide (Mansoor et al., 2003; Briddon, 2003; Liao et al., 2007; Sattar, 2025). Although geminiviruses are primarily recognized as plant-infecting pathogens, several lines of evidence suggested certain geminiviruses possess cross-kingdom replication and gene expression capabilities in both eukaryotic and prokaryotic systems, as described below.

The accumulation of replicative form DNAs of a geminivirus, tomato leaf curl virus (ToLCV), was first observed in *Agrobacterium tumefaciens* (Rigden et al., 1996), demonstrating that ToLCV DNA replication was supported by the bacterial machinery. Similar phenomenon was later reported for other geminiviruses, tomato yellow leaf curl virus (TYLCV) and African cassava mosaic virus (ACMV), in both *A. tumefaciens* and *E.coli* (Selth et al., 2002). The promoter activities of all or part of the ToLCV-encoded genes in *A. tumefaciens* or *E. coli*, respectively, were also demonstrated using a β-glucuronidase reporter system (Selth et al., 2002). In our previous work, we have also observed the production of genomic sscDNAs of another geminivirus, ageratum yellow vein virus (AYVV) in *E. coli* harboring a construct consisting of unit-length AYVV genome cloned in a M13-based vector (Wu et al., 2007). The phenomenon was dependent on the presence of an open reading frame encoding the viral replication associated protein (Rep) in the construct, suggesting that the Rep protein could be actively expressed in *E. coli*. In addition, we have previously identified and characterized a cryptic promoter with the core region mapped at nucleotide positions 762–869 in the AYVV genome, designated as AV3 promoter. This AV3 promoter exhibits constitutive activity capable of driving the expression of downstream green fluorescent protein (GFP) reporter gene both in yeast (*Saccharomyces cerevisiae*) and *E. coli* (Wang et al., 2013; 2014). The above observations indicated that certain geminiviruses are capable of gene expression and genome replication in hosts belonging to two different domains, *Eucarya* and *Bacteria*, and supported the hypothesis of prokaryotic origins of geminiviruses (Koonin and Ilyina, 1992; Krupovic et al., 2009; Kazlauskas et al., 2019). However, the underlying mechanism for gene expression and DNA replication in prokaryotic systems remained to be elucidated.

**Fig. 1:**
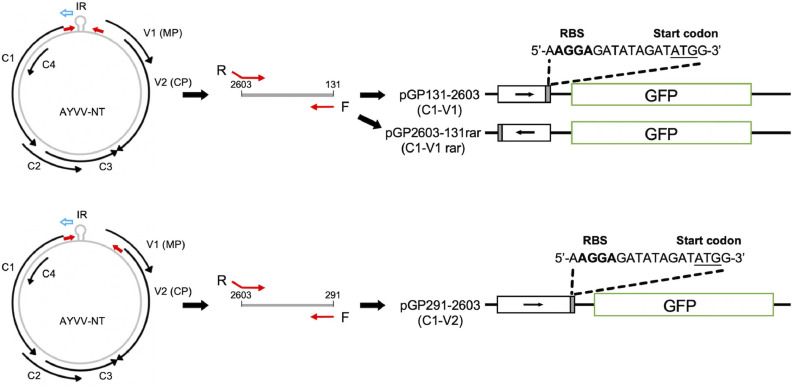
Cloning of IR-containing fragments from AYVV-NT into the pGlow-TOPO® vector. Schematic diagrams show the genomic organization of AYVV-NT and the positions of the cloned fragments. Upper panel, IR-containing fragments C1-V1 (nt 131–2603) and C1-V2 (nt 291–2603) were cloned into the pGlow-TOPO® vector in the c-sense orientation, generating pGP131–2603 and pGP291–2603, respectively. A construct (pGP2603–131 rar), containing the C1-V1 fragment in the reversed orientation, was also selected during screening and used as a negative control in the following experiments. Black curved arrows represent annotated ORFs of AYVV-NT. The blue hollow arrow indicates the c-sense orientation. Red arrows denote PCR primers used for amplification. Cloned fragments are shown as open boxes, with arrowheads indicating their orientation relative to the c-sense strand. Ribosome binding sites (RBS; bold letters) and start codons (underlined) were introduced upstream of GFP open reading frame. The label 'rar' refers to an artificial RBS positioned in the reverse orientation relative to the GFP coding sequence.

The sscDNA genomes of geminiviruses are ambisense, and the transcription of genes in both virion-sense (v-sense) and complementary-sense (c-sense) directions is thought to be initiated from the promoter-containing region located between the C1 and V1 genes, designated as intergenic region (IR) (Fig. 1). The c-sense genes are early genes as they encode proteins required for S-phase progression and replication initiation (Rep, or C1), transcription activation of v-sense genes (TrAP, or C2), replication enhancement (REn, or C3), and suppression of gene silencing (C4) (Fondong, 2013; Borah et al., 2016; Devendran et al., 2022). On the other hand, the v-sense genes are late genes that provide functions for viral movement (MP, or V1) and encapsidation (CP, or V2) that are required at the later stages of infections. The promoter activities and regulatory mechanisms of geminiviruses have been extensively studied in eukaryotic systems, by using transgenic plants and protoplasts (Zhan et al., 1991; Eagle et al., 1994; Eagle and Hanley-Bowdoin, 1997; Dry et al., 2000; Frey et al., 2001; Shivaprasad et al., 2005; Sunter and Bisaro, 1997; 2003). However, the corresponding promoters and regulatory elements, and possible roles of different host species or strains, have not been explored in the prokaryotic systems.

Therefore, in the present study, we aimed to characterize the promoters and regulatory elements located within the IR of a representative geminivirus, AYVV isolate NT (AYVV-NT) in a prokaryotic context. Using a promoter trapping vector system with GFP reporter, we evaluated promoter activities in both directions, and fine-mapped the corresponding promoter cores through a series of deletion mutants. The results offered further insights into the regulatory mechanisms for geminivirus gene expressions in prokaryotic systems, and established the basis for exploration of potential applications in biotechnology.

## 2 Materials and methods

### 2.1 Virus isolate and bacteria strains

A previously characterized isolate of AYVV collected in Nan-Tou County, Taiwan, designated as AYVV-NT (Wang et al., 2013; 2014), was used in this study for the characterization of promoter activities in the IR in the prokaryotic system. The unit-length genome of AYVV-NT has been cloned into the *Bam*HI restriction enzyme site of the pUC119 vector. The complete sequence has been determined (GenBank accession number EF458639, Wu et al., 2007), and the construct was designated pAYVV-NT.

To analyze the influence of different prokaryotic systems on AYVV promoter activities, three strains of *E. coli*, namely DH5α (Invitrogen, Taiwan), BL21(DE3) (Invitrogen, Taiwan), and TOP10 (Invitrogen, Carlsbad, CA, USA), were used in this study. All bacteria were cultivated in Lysogeny Broth (LB) liquid media, or plated on LB containing 3 % (w/v) agar, at 37 °C for 16 hrs following standard protocols.

### 2.2 Plasmid construction

The intergenic region (IR, corresponding to nucleotides 2603 to 131, Fig. 1) of AYVV-NT was subcloned into the pGlow-TOPO® vector using the TOPO® Reporter kit (Invitrogen, life technologies, Carlsbad, CA, USA). This promoter-trapping vector contains green fluorescent protein (GFP) and lacks a prokaryotic ribosomal binding site (RBS) upstream of its reporter, originally designed for analyzing promoter activity in mammalian cells. To facilitate GFP translation in prokaryotes, a prokaryotic ribosome binding site (RBS), a spacer, and a translational initiation codon, ATG, were artificially added upstream to the open reading frame (ORF) of the GFP reporter gene, in the reverse primer (C1-V1 R, sequence: 5′-CCATATCTATATCTCCTTTTGACTTGGT-CAATCGG-3′) following manufacturer’s instructions. For PCR amplification, the reverse primer targeting the AYVV-NT intergenic region (C1-V1 R) (Wang et al., 2013), was used in conjunction with specific forward primers for the C1-V1 and C1-V2 fragments (Table 1). The cloning strategy and the orientations of the IR in various constructs are depicted in Fig. 1. PCR amplification was performed under the following thermal cycling conditions: initial denaturation at 94 °C for 5 min; followed by 35 cycles of 94 °C for 30 s, 52 °C for 30 s, and 72 °C for 30 s; and completed with a final extension at 72 °C for 5 min. PCR products were purified by ethanol precipitation and ligated into the pGlow-TOPO® promoter-trap vector according to the manufacturer’s instructions. The resulting constructs were transformed into chemically competent *E. coli* TOP10 cells and plated on selective LB medium. After incubation at 37 °C for 16 hrs, colonies harboring putative IR fragments were screened by colony PCR using the same cycling parameters described above. Clones harboring plasmids with inserts of expected size were subsequently verified by nucleotide sequencing. The AV3 core promoter of AYVV-NT characterized in our previous studies (Wang et al., 2013; 2014), corresponding to nt 764–871 of AYVV-NT genome, was cloned into the pGlow-TOPO® vector, referred to as AYVV-AV3 promoter, and used as a positive control in this study.

**Table 1:** List of primers used in this study.

Primer name	Sequence (5′ → 3′)^1^	Purpose^2^
C1-V1 promoter F	GTTTAAAATAATACTTGGAGAATAAG	A, D
C1-V2 promoter F	AATTGCGAGCACGAATTACTGAAATT	A
C1-V1 R	CCATATCTATATC**TCCT**TTTGACTTGGTCAATCGG	A
nt111–90 F	AATAAGTATAAATAGGGGACCA	B, E
nt91–70 F	CACTTAATAATTTAACTTTGAG	B, E
nt71–54 F	AGGGAGCGTTCTGAGTGG	B, E
nt53–34 F	GGGATTTTTGTTCGTGGTAG	B, E
nt34–12 F	GGACCACTTTTAAAAAAATCGC	B, E
nt11–2742 F	GCGGCCATCCGGTAATATTATACGG	B, E
nt2746–2727 F	TACGGATGGCCGCTTTTGGA	B, E
nt2726–2699 F	TTTCAATTTAAAGTATGAATTTCTTTTC	B, E
Inverse R	AAGCTTAAGTTTAAACGCTAGC	B, E, F
Inverse F	A**AGGA**GATATAGATATGGAAGGGCAATTCTGCAG	C, D, E
nt2646–2673 R	TGTACTGGAGTCCTATATATAGTTAGAC	C, F
nt2671–2699 R	GACATCAAATGGCAATTATTGTAATTTTG	C, F
nt2699–2729 R	GAAAAGAAATTCATACTTTAAATTGAAATCC	C, F
nt2728–2747 R	CCAAAAGCGGCCATCCGTAT	C, F
2728–10 c-sense-F	GCCATCCGGTAATATTATACGGATGGCCGCTTTTG GA**AGGA**GATATAGATATGGA	G
2728–10 c-sense-R	CCATATCTATATC**TCCT**TCCAAAAGCGGCCATCCG TATAATATTACCGGATGGCA	G
colony PCR R-2	CAGAATTGCCCTTCCATATCTATATCTC	H
dxs-F	CGAGAAACTGGCGATCCTTA	I
dxs-R	CTTCATCAAGCGGTTTCACA	I
GFP-F	ATGGCTAGCAAAGGAGAAGAAC	J
GFP-R	GGGTAAGCTTTCCGTATGTAGC	J

1 Bold letter mark the RBS, which is the reverse complement of AGGA, and the start codon (ATG) is highlighted with an underline.

2 The primary purpose of each primer is categorized as follows. A, for construction of the full-length IR in the c-sense orientation; B, for generation of 5′ unidirectional deletion mutants in the c-sense orientation; C, for generation of 3′ unidirectional deletion mutants in the c-sense orientation; D, for construction of the full-length IR in the v-sense orientation; E, for generation of 3′ unidirectional deletion mutants in the v-sense orientation; F, for generation of 5′ unidirectional deletion mutants in the v-sense orientation; G, for construction of the nt 2728–10 fragment in the c-sense orientation; H, for colony PCR screening the constructs in both orientations; I, for internal control of qPCR and RT-qPCR; J, for target gene of qPCR and RT-qPCR.

### 2.3 Identification of core promoter sequence in the IR through directional deletion

**Fig. 2:**
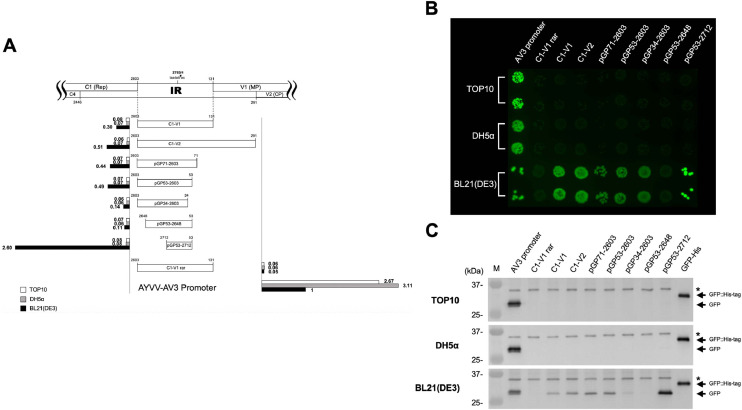
Identification of the promoter and regulatory regions in the c-sense orientation of the IR. (A) Schematic representation of various IR-derived promoter constructs fused upstream of a GFP reporter gene in the c-sense orientation. The positions of the 5′ and 3′ deletion endpoints are indicated relative to the AYVV-NT genome. Above the tested promoter constructs, a diagram shows the organization of IR-containing regions, including flanking rightward- and leftward-oriented ORFs (outlined boxes) and the conserved nonanucleotide (TAATATTAC) at the junction of nt 2753 and 1. The bar graph presents relative GFP fluorescence intensities measured in three *E. coli* strains: TOP10 (white bars), DH5α (gray bars), and BL21(DE3) (black bars). Fluorescence values were normalized to the concentration (OD_600_) of each culture and expressed as folds relative to that of the AV3 promoter activity in BL21(DE3), which was defined as 1.0 and used as the reference. (B) Visualization of GFP expression in colonies. UV fluorescence images of *E. coli* colonies harboring each construct were captured following incubation at 37 °C for 16 hrs. Colonies were exposed to long-wavelength (365 nm) UV light to detect GFP signal. Data corresponded to constructs and *E. coli* strains are shown in panel A. (C) Western blot analysis of GFP expression from c-sense promoter constructs in various *E. coli* strains. Total cellular proteins were extracted from bacteria harboring individual constructs as indicated on top of each lane, separated by electrophoresis through a 12.5 % polyacrylamide gel containing 1 % SDS, transferred to a PVDF membrane, and probed with anti-GFP serum. The upper, middle, and lower panels correspond to samples from *E. coli* TOP10, DH5α, and BL21(DE3), respectively. Lane M contains molecular weight markers with sizes (in kDa) indicated on the left. The black arrows on the right indicate the expected molecular weights of GFP and the positive control His-tag-GFP fusion protein at 27 kDa and 29.9 kDa, respectively, as calculated from the amino acid sequences. A non-specific band is denoted by an asterisk (*).

To identify the core promoter region within the IR, a series of unidirectional deletions were introduced via inverse PCR following the method of Ochman et al. (1988). Both the c- and v-sense promoter elements were fine-mapped with deletions from the 5′- and 3′-termini of the IR. For mapping the c-sense promoter core region, plasmids containing the full-length IR (C1-V1) in the c-sense orientation were used as templates for inverse PCR. For deletions in the direction from C1 gene to V1 (Fig. 2A), individual forward primers (Table 1), each targeting a specific upstream region, were used along with the common reverse primer Inverse R (Table 1), with each construct removing approximately 20 nucleotides. The resulting 5′-truncated plasmids were subsequently used for deletions from the 3′-terminus, employing reverse primers in combination with the common forward primer Inverse F (Table 1). In parallel, for construction of plasmid harboring the IR in the v-sense orientation, a plasmid containing the C1-V1 fragment cloned in the reverse direction into the pGlow-TOPO® vector (designated C1-V1 rar) was used as the template. An RBS and a start codon in frame with the vector-borne GFP ORF were engineered using an inverse PCR-based strategy. Subsequent unidirectional deletions from both 5′- and 3′-termini were carried out in the same manner as described above, using primer sets listed in Table 1. All PCR reactions were performed under identical cycling conditions: initial denaturation at 94 °C for 5 min; followed by 25 cycles of 94 °C for 30 s, 52 °C for 30 s, and 72 °C for 6 min; and completed with a final extension at 72 °C for 5 min. PCR products were self-ligated and introduced into *E. coli* DH5α through the CaCl_2_ transformation method (Sambrook and Russell, 2001). Colonies were selected and verified by sequencing as described above.

### 2.4 Assessment of promoter strength via GFP fluorescence

Promoter activity was assessed by quantifying GFP fluorescence using an FLx800™ Multi-Detection Microplate Reader (BioTek Instruments, Winooski, VT, USA), following the method of Wang et al. (2013). Recombinant plasmids were transformed into *E. coli* strains DH5α, BL21(DE3), and TOP10, as described above. For GFP fluorescence measurement, a single colony was cultured in 2 ml LB medium containing ampicillin (100 μg/ml) at 37 °C for 16 hrs. After incubation, 200 μl of each culture was transferred into a black, flat-bottom 96-well plate in triplicate for fluorescence measurement (excitation wavelength: 400 nm; emission wavelength: 508 nm; sensitivity: 60) using Gen5 software (BioTek Instruments, Winooski, VT, USA). Fluorescence values were normalized to the bacterial culture concentrations (determined by OD_600_) of each culture and expressed as folds of the intensities relative to that of the AV3 promoter in BL21(DE3), which served as the positive reference, as the measurement of promoter activities.

### 2.5 Quantification of plasmid copy number in *E. coli* by real-time quantitative PCR (qPCR)

To assess whether the observed differences in promoter activity were attributable to variations in plasmid copy number among constructs in different *E. coli* strains, real-time qPCR was performed. All strains were cultured under identical conditions as described above. A single colony was cultured in 2 ml LB medium containing ampicillin (100 μg/ml) at 37 °C for 16 hrs. Cultures were then normalized to an OD_600_ of 1.0, and 1 ml of each adjusted culture was harvested for total DNA extraction. Genomic DNA was isolated using the Geno Plus™ Genomic DNA Extraction Miniprep System Kit (VIOGENE, Taiwan), following the manufacturer’s instructions for bacterial cells. The prepared DNA samples were used as templates for qPCR along with specific primers (as listed in Table 1) and iTaq Universal SYBR® Green Supermix (BIO-RAD), according to the manufacturer’s guidelines. qPCR amplification and analysis were performed using a LightCycler instrument (Roche Diagnostics) with LC480 software v1.5.0. Plasmid copy number was calculated using the ΔΔCt method. Primers targeting the chromosomal *dxs* gene were used as an internal reference to quantify plasmid copy number relative to bacterial genomic DNA, which was assumed to be present at one copy per cell (Lee et al., 2006). To ensure reproducibility, three independent biological replicates were performed for each assay, and each biological replicate was analyzed using three technical replicates in the qPCR analysis.

### 2.6 Quantitation of the GFP mRNA using reverse transcription qPCR (RT-qPCR)

For transcript-level analysis, bacterial cultures were grown, normalized, and harvested as described above. Total RNA was extracted from 1 ml of each OD_600_-normalized culture using the VeZol-pure total RNA isolation Kit (Vazyme, Nanjing, Jiangsu Province, China), according to the manufacturer’s protocol. The extracted samples were treated with RQ1 RNase-Free DNase (Promega, Madison, WI, USA) to remove residual genomic DNA contamination. Reverse transcription was performed using the GoScript™ reverse transcription system (Promega, Madison, WI, USA) and gene-specific reverse primers targeting *gfp* or *dxs*, following the manufacturer’s guidelines to synthesize the first-strand cDNA. Control reactions lacking reverse transcriptase were included to confirm the absence of genomic DNA contamination. qPCR reactions were performed using the above cDNA diluted 10-fold (3 µl) as template, and amplification conditions, data acquisition, and analysis were identical to those described above. Relative transcript levels were calculated using the ΔΔCt method, with *dxs* serving as the internal reference gene. Replication and qPCR analysis were conducted as described for plasmid copy number determination.

### 2.7 Colony-based GFP fluorescence assay

To visualize GFP fluorescence in bacterial colonies, *E. coli* cultures prepared as described above were diluted 20,000-fold in fresh LB, and 2 μl of each diluted sample were spotted in duplicate onto LB agar plates supplemented with 100 μg/ml ampicillin. Plates were then incubated at 37 °C for 16 hrs and subsequently photographed using the LAS-4000 luminescent/fluorescent imaging system (Fujifilm Life Science) to document GFP fluorescence. Fluorescence excitation was performed with a blue LED source with an excitation wavelength of 480 nm, and emission was detected using a filter centered at 510 nm. Image acquisition was conducted under optimized exposure conditions.

### 2.8 Western blot analysis of GFP expression

To confirm GFP expression, immunoblotting was conducted with GFP-specific antibody (Wang et al., 2013). Bacterial clones were cultured under identical conditions as described, and culture densities (OD_600_ values) were recorded for normalization with the respective GFP fluorescences. Equal volumes (40 μl) of each culture were mixed with 5X SDS sample buffer, boiled at 100 °C for 5 min, and resolved by electrophoresis through a 12.5 % polyacrylamide gel containing 1 % SDS. Proteins were transferred to a polyvinylidene difluoride (PVDF) membrane (Millipore, USA) and incubated with laboratory-generated primary antiserum from rabbits against GFP (Kuo et al., 2018) at a 1:5000 dilution, followed by alkaline phosphatase-conjugated goat anti-rabbit IgG (1:5000; SIGMA-Aldrich, St. Louis, MO, USA). Detection was performed using nitro-blue tetrazolium (NBT) and 5‑bromo-4‑chloro-3-indolyl phosphate (BCIP) colorimetric substrates (Promega, Madison, WI, USA).

### 2.9 Computer-aided identification of promoter features within the IR

Putative promoter elements within the IR were analyzed using multiple web-based tools. The positions of the −10 and −35 elements were determined via the phiSITE database (http://www.phisite.org/main/) (Klucar et al., 2010). Additional analyses of −10 and −35 motifs and transcription start sites (TSSs) were conducted using the SAPPHIRE.CNN platform (http://sapphire.biw.kuleuven.be) (Coppens and Lavigne, 2020). Factors-associated promoter sequences within the IR were identified using the iPromoter-2 L server (http://bliulab.net/iPromoter-2L/server) (Chen et al., 2014, 2015; Liu et al., 2018).

## 3 Results

### 3.1 Characterization of promoter activities within IR in different *E. coli* strains

It has been proposed that geminiviruses initiate early gene expression in the c-sense orientation upon host infection, particularly for replication-associated protein (Rep) (Borah et al., 2016). To determine whether the IR of AYVV-NT exhibits promoter activity in c-sense, two DNA fragments were cloned: one flanked by the upstream regions of the C1 and V1 ORFs, and another including the upstream regions of C1 and V2 ORFs—designated as C1-V1, C1-V2, respectively (Fig. 1). All primers used for PCR amplification and construct generation are summarized in Table 1 and the cloning strategy is illustrated in Fig. 1. Each PCR product contained an 18-nt artificial sequence with an RBS (-AGGA-) and initiation codon (ATG) in-frame and adjacent to GFP ORF in the c-sense direction. Additionally, as the cloning strategy allows the insertion of PCR fragment in both orientations, a construct containing the C1–V1 fragment inserted in the reverse orientation, designated C1–V1 rar (for reversed artificial RBS), was selected and used as an experimental negative control. Due to this inverted insertion, the RBS and start codon were oriented in the opposite direction relative to the GFP coding sequence, thereby preventing GFP expression (Fig. 1).

**Fig. 3:**
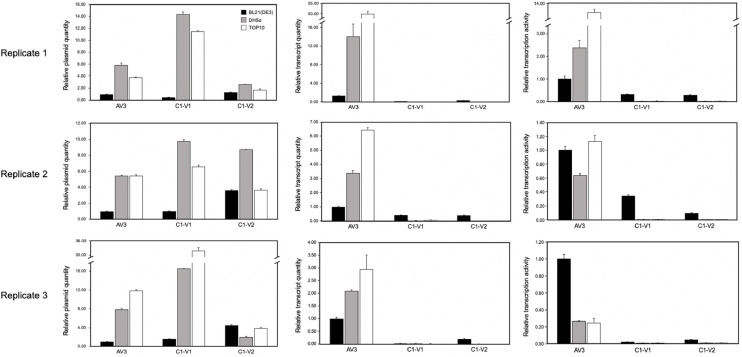
Quantitative analysis of transcriptional activity in different *E. coli* strains by qPCR and RT-qPCR. Relative plasmid quantity (left panels), relative transcript quantity (middle panels), and relative transcriptional activity (right panels) were measured for AV3, C1-V1, and C1-V2 promoter constructs in *E. coli* BL21(DE3) (black), DH5α (gray), and TOP10 (white). Data are shown for three independent biological replicates (Replicate 1–3, top to bottom). Relative values were normalized to the corresponding quantity in *E. coli* BL21(DE3) harboring AV3 promoter within each replicate. Error bars represent the mean ± standard deviation from three experiments. Broken y-axes are used to accommodate differences in magnitude across conditions.

All promoter constructs were transformed into *E. coli* BL21(DE3), DH5α, and TOP10. GFP expression was initially assessed under UV illumination. Preliminary results revealed that both C1–V1 and C1–V2 exhibited promoter activity in the c-sense orientation, albeit detectable fluorescence was exclusively observed in BL21(DE3) (Fig. 2B). To verify these findings, further analyses were conducted with quantitative fluorescence analysis using a FLx800™ Multi-Detection Microplate Reader and western blot analysis with GFP-specific antibodies. The average fluorescence intensity driven by the AV3 promoter in BL21(DE3) was used as the reference for normalization, and the relative expression levels of all other constructs in the three *E. coli* strains were calculated accordingly. The AV3 promoter showed detectable activity across all three strains, although at different expression levels. In contrast, C1–V1 and C1–V2 drove GFP expression exclusively in BL21(DE3), with no detectable activity in DH5α or TOP10. Western blot analysis corroborated the UV examination results, with GFP detected solely in BL21(DE3) (Fig. 2C). To determine whether these strain-dependent differences arose from variations in transcriptional activity, relative plasmid copy number and relative transcript abundance were quantified by qPCR and RT-qPCR (Fig. 3), respectively. Relative transcript levels were first normalized to plasmid copy number (representing transcription activities) and subsequently scaled by setting the AV3 promoter transcription activity in BL21(DE3) to 1. The results revealed that, consistent with the protein expression pattern, transcription driven by C1-V1 and C1-V2 was restricted to BL21(DE3), whereas the AV3 promoter showed higher transcriptional activity than C1-V1 and C1-V2 across all three strains. Notably, AV3-driven transcription also varied substantially among biological replicates, indicating pronounced transcriptional heterogeneity within isogenic cell populations. This observation is in agreement with previous studies showing that promoter activity, defined by the flux of RNA polymerase per second per DNA (RNAP s^-1^ DNA^-1^), can be highly dispersed among individual *E. coli* cells under identical conditions (Shao et al., 2021). These results indicated that the IR of AYVV-NT possesses functional c-sense promoter activity, particularly evident in BL21(DE3) (Fig. 2A). Taking this into account, subsequent experiments were performed using the three strains of *E. coli*.

### 3.2 Core region mapping of the C-sense promoter in the IR

**Fig. 4:**
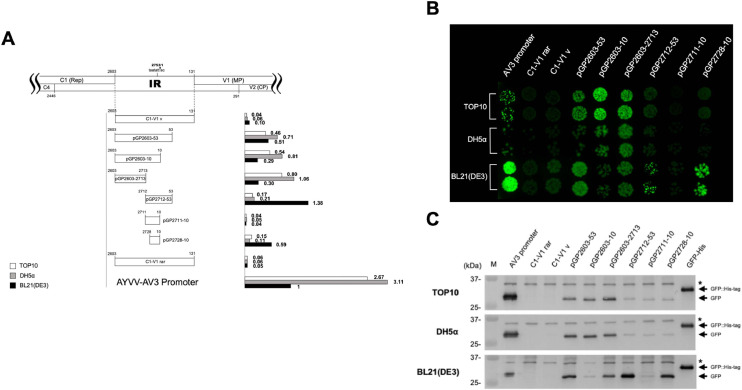
Identification of the core promoter region in the v-sense direction of the IR. (A) Schematic representation of the constructs containing various IR-derived fragments fused to the GFP reporter gene in the v-sense orientation. The 5′ and 3′ endpoints of each construct are indicated relative to the AYVV-NT genome. Annotations are consistent with those in Fig. 2. (B) Visualization of GFP expression from each construct under long-wavelength UV light following incubation at 37 °C for 16 hrs. Constructs were transformed into *E. coli* strains TOP10, DH5α, and BL21(DE3), and the resulting colony fluorescence corresponded to the constructs and strains as shown in panel A. (C) Western blot analysis of GFP expression from v-sense promoter constructs in *E. coli* strains. Experimental procedures and labeling conventions are consistent with those in Fig. 2.

To identify the core region of the promoter located in the c-sense, an inverse PCR-based a series of unidirectional deletion mutants were generated. This strategy enabled systematic truncation of the promoter sequence from either the 5′- or 3′-terminus to delineate the minimal region necessary for transcriptional activity, i.e., the promoter core. Preliminary promoter activity assays indicated that the two constructs, C1–V1 and C1–V2, showed no significant differences in GFP expression, thus the construct C1–V1 was chosen as the template for detailed promoter dissection. As an initial step, 5′-terminus deletion analysis was performed. When the 5′ region was truncated down to nt 53, the promoter retained detectable activity, although with a slightly decrease in GFP expression levels compared to that of the full-length fragment. Further deletion to nt 34 resulted in a marked decline in expression. Both microplate-based fluorescence quantification and western blot analysis confirmed this reduction (Fig. 2A, C); however, under UV illumination, visible green fluorescence signal was still observed, indicating that GFP accumulation remained sufficient for detection by the naked eye (Fig. 2B). These results suggest that the region spanning nt 34–53 contains critical cis-acting elements required for maintaining robust promoter activity and was therefore preserved in subsequent experiments. To continue defining boundaries of the core promoter, 3′-terminus deletions were carried out using pGP53–2603 construct as the template. When the 3′ region was truncated to nt 2648, promoter activity was significantly reduced. Interestingly, further deletion to nt 2712 resulted in a marked increase in promoter activity (Fig. 2). Both fluorescence analyses and western blot analysis (Fig. 2) revealed that promoter strength in the c-sense orientation was only detectable in *E. coli* BL21(DE3), while no significant activity was observed in DH5α or TOP10. However, a faint GFP signal was detected in DH5α when the promoter fragment was shortened from nt 2603–53 to nt 2712–53 (Fig. 2C and Fig. 4C). These results defined the core promoter region in the c-sense orientation as spanning nt 2712–53.

### 3.3 Mapping of the core promoter region in the V-sense orientation of IR

Given the ambisense nature of geminivirus genomes, proteins can be encoded in both the c-sense and v-sense orientations. Consequently, it is expected that the promoter located in the intergenic region possesses bidirectional activity. Based on this rationale, we used the non-fluorescent negative control plasmid ‘C1–V1 rar’ as the template for generating the v-sense promoter construct via inverse PCR. The resulting construct was engineered to include an RBS and an ATG codon in frame to the GFP coding region, followed by a series of unidirectional deletion analyses to identify core promoter regions.

In the v-sense orientation, we generated a construct with the same inserted sequence spanning nt 2603–131 as C1–V1, designated as C1–V1 v. Upon transformation, GFP accumulation was detected exclusively in *E. coli* BL21(DE3) (Fig. 4). Unexpectedly, shortening the 3′-terminus to nt 53 led to detectable GFP expression in all three strains—BL21(DE3), DH5α, and TOP10—indicating that the v-sense promoter can be functionally recognized by transcriptional machinery across different *E. coli* backgrounds (Fig. 4). This observation was further confirmed by western blot analysis (Fig. 4C). Further 3′ deletion down to nt 10, the GFP accumulation remained relatively unchanged in DH5α and TOP10 but decreased significantly in BL21(DE3). Interestingly, modifying the 5′-terminus to nt 2713 elevated GFP accumulation in DH5α and TOP10, while BL21(DE3) showed no significant change.

Using the nt 2603–53 fragment, which exhibited activity in all three strains, as the template for additional 5′ deletions, GFP accumulation was significantly decreased in DH5α and TOP10 when the upstream sequence was limited to nt 2712. In contrast, the construct exhibited a marked increase in GFP expression in BL21(DE3) (Fig. 4). The resulting nt 2712–53 fragment corresponds in length to the c-sense construct pGP53–2712, despite operating in opposite orientations. Both constructs showed enhanced GFP accumulation at this size in BL21(DE3), suggesting that the core promoter region may be present within this interval. Moreover, the longer region from nt 2603–2713 also exhibited promoter activity across all strains, implying the presence of functional elements such as −10 and −35 boxes that are capable of being recognized by bacterial σ factors, thereby enabling RNA polymerase binding and transcription initiation of downstream GFP gene (Fig. 4A).

To further define the essential sequence within the 2712–53 region, pGP2712–53 was used as the template for additional truncation. Both fluorescence and western blot analyses showed a pronounced decrease in GFP expression when the 3′ boundary was set at nt 10. However, constructs retaining the region from nt 2728 to 10 maintained significant promoter activity in BL21(DE3) (Fig. 4), allowing us to narrow down the v-sense core promoter region to a 36-nt sequence from nt 2728 to 10.

### 3.4 Comparative analysis of bidirectional promoter activities using identical IR-Derived fragments in c- and v-sense orientations

**Fig. 5:**
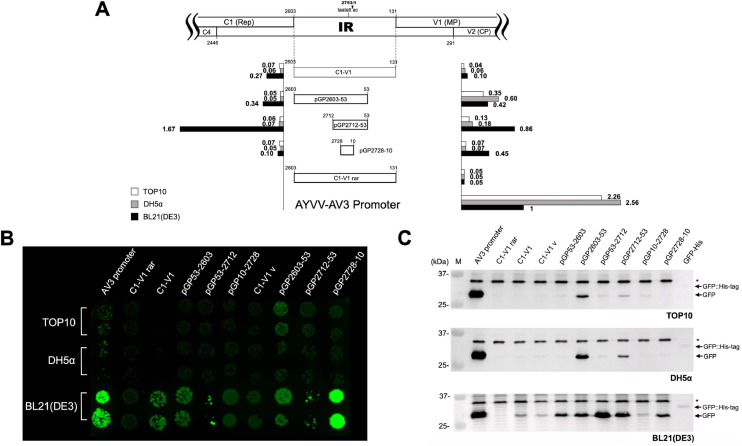
Comparative analysis of bidirectional promoter activity using IR-derived fragments of identical length. (A) Schematics of constructs containing IR fragments of equal length fused to the GFP reporter gene in either the c-sense or v-sense orientation. Promoter activities were assessed in *E. coli* strains TOP10, DH5α, and BL21(DE3), as described in Fig. 2. Annotations follow the same conventions as in Fig. 2. (B) UV fluorescence images of *E. coli* colonies harboring the constructs shown in panel A. Images were captured following incubation at 37 °C for 16 hrs under long-wavelength (365 nm) UV light. (C) Western blot analysis of GFP expression in *E. coli* strains harboring the constructs depicted in panel A. Experimental procedures and labeling conventions are consistent with those in Fig. 2.

Since the nt 2728–10 fragment exhibited promoter activity in the v-sense, we sought to determine whether it also functioned in the c-sense direction. To test this, the fragment was cloned into the pGlow-TOPO vector in the c-sense orientation and transformed into three *E. coli* strains. The results of promoter activity assays confirmed that the fragment indeed retained promoter activity in the c-sense as well. Thus, we defined the core promoter elements of the AYVV-NT IR to be located within nt 2728–10. To assess directional and strain-specific differences in promoter activity, the constructs harboring IR-derived fragments through unidirectional deletion analysis used for promoter core mapping were analyzed for comparison of promoter activities in both c-sense and v-sense orientations. These IR-derived fragments, including nt 2603–131 (C1–V1 and C1–V1 v), nt 2603–53 (pGP53–2603 and pGP2603–53), and nt 2712–53 (pGP53–2712 and pGP2712–53), were systematically analyzed for promoter activities by microplate-based fluorescence quantification and western blotting (Fig. 5).

Since the nt 2603–131 region encompasses the full IR sequence, promoter activity from this construct was considered as the baseline. Based on the results, the c-sense exhibited higher baseline promoter activity than did the v-sense, consistent with previous observations by Selth et al. (2002). It is worth noting that when the IR fragment was shortened from nt 131 to 53, a notable increase in v-sense promoter activity was observed. This may suggest the removal of potential repressor binding sites (operators) between nt 131 and nt 53 that hinder RNA polymerase access, thereby enhancing transcription. In the nt 2712–53 fragment, both orientations showed enhanced promoter activity, with a greater increase in the c-sense direction.

### 3.5 In silico prediction of promoter motifs within the AYVV-NT intergenic region

**Fig. 6:**
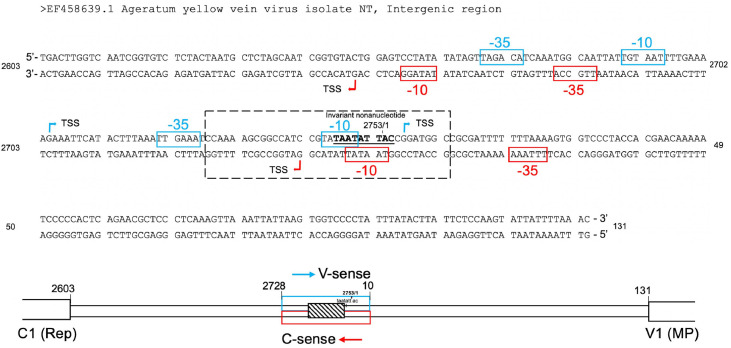
Mapping of the prokaryotic promoter consensus sequences and a bidirectional core element within the IR. Nucleotide sequence of the IR from ageratum yellow vein virus isolate NT (AYVV-NT, GenBank accession No. EF458639.1), annotated with promoter-associated motifs in both the virion-sense (v-sense; blue) and complementary-sense (c-sense; red) orientations. Computer-predicted putative −10 and −35 elements are boxed and color-coded accordingly. Transcription start sites (TSSs), inferred using promoter analysis tools, are indicated by bent arrows. A dashed box highlights a central region containing an invariant nonanucleotide motif (TAATATTAC), flanked by predicted TSSs and associated promoter motifs on both strands. The diagram at the bottom illustrates the genomic context of the IR, located between the C1 (Rep) and V1 (MP) open reading frames. Arrows indicate transcriptional directionality in both orientations, and the hatched box corresponds to the regulatory core region depicted in the sequence above. Nucleotide positions are numbered accordingly.

The above finding prompted us to revisit our promoter element analysis using web-based tools. In the v-sense orientation, two sets of predicted −10 and −35 elements had previously been identified. Similarly, computer-aided promoter prediction revealed corresponding −10 and −35 motifs in the c-sense direction within the deleted nt 2603–2713 region (Fig. 6). These results suggest that the presence of RNA polymerase recognition elements in both orientations may lead to transcriptional interference or competition. Consequently, when one set of promoter elements is removed, RNA polymerase binding becomes more specific, leading to improved transcriptional efficiency in the remaining direction.

In prokaryotic systems, transcription initiation requires the recognition of conserved promoter elements located upstream of the transcription start site—specifically the −10 (Pribnow box) and −35 regions—by the σ factor, which subsequently recruits RNA polymerase to the promoter for transcription initiation (Hawley and McClure, 1983; Ross et al., 1993). Fig. 6 illustrates the identified multiple putative −10 and −35 elements, along with transcription start sites (TSSs), in both the c-sense and v-sense orientations within the intergenic region of AYVV-NT. Notably, in the nt 2603–2713 fragment, one set of −10 and −35 motifs was predicted for each orientation. A similar pattern was observed in the nt 2712–53 fragment, indicating that both fragments possess promoter features in both directions. These predictions are consistent with our experimental findings, where the nt 2603–2713 fragment in the v-sense exhibited promoter activity across all three tested *E. coli* strains (Fig. 4). Furthermore, analysis of the 36-nt minimal promoter fragment (nt 2728–10), which retained promoter activity in both orientations, revealed that only a −10 motif was present in both the c-sense and v-sense directions. This region is AT-rich, a characteristic commonly associated with bacterial promoters, and the observation suggests that the core promoter elements in both orientations may partially or completely overlap. Collectively, these findings support the presence of a shared bidirectional core promoter within the IR of AYVV-NT, capable of initiating transcription in both the c-sense and v-sense orientations.

## 4 Discussion

Previous studies have demonstrated that geminiviruses are capable of replicating in prokaryotic systems such as *A. tumefaciens* and *E. coli*, and that promoters located upstream of all individual viral genes are transcriptionally active in *A. tumefaciens*, but only 2 of the c-sense genes (C1 and C2) were active in *E. coli* (Rigden et al., 1996; Selth et al., 2002), suggesting the involvement of different bacterial transcriptional machineries. We have also observed the formation of unit-length sscDNA of AYVV in *E. coli* harboring a phage M13-based vector containing AYVV genome (Wu et al., 2007). In this study, the promoter core and associated regulatory elements in IR were identified and characterized in different strains of *E. coli*, which offered further insight into the activation and regulation of expressions of geminivirus genes in prokaryotic systems.

In addition, the results in this study also provide hints on possible reasons underlying the retention of sequence elements recognizable by prokaryotic systems within the genomes of plant-infecting geminivirses over the course of evolution. Since most of the plant cells are terminally differentiated and arrested in G_0_/G_1_ phase of cell cycle, which do not support the DNA synthesis or replication functions, except for cells in the epical meristem layer or cambium cells. Thus, most of the cellular environments are not suitable for geminivirus replication at the initial stage of infection. The progression of geminivirus-infected cells from G_0_/G_1_ phase to S phase is dependent on the function of viral Rep proteins, which interact with retinoblastoma-related protein (RBR protein) to release the E2F transcription factor required for the expression of S phase genes (Gutierrez, 2000). The prokaryotic-like organelles, e.g., chloroplasts and mitochondria, with possible evolutionary origins from endosymbiotic bacteria in plant cells (Bendich, 2007; Allen, 2015; Rose, 2019), may provide appropriate environments for the expression of viral Rep gene driven by the prokaryotic promoter regions located in the IR at the initial stage of infections. Compared to the nucleus, many copies of chloroplasts or mitochondria may exist in a single cell (Bendich, 2007; Rose, 2019), providing sufficient subcellular locations for the expression of early viral genes. In addition, the DNA synthesis regulation in chloroplasts and mitochondria can be uncoupled from the cell division regulation for nuclear DNA replication (Bendich, 2007; Rose, 2019), these organelles might also support the function for converting the invading viral sscDNA into the double-stranded DNA for the expression of Rep protein at the early stage of infections.

The gene expression in the c- and v-sense of geminiviruses is temporally regulated, indicating differential promoter control upstream of the *C1* (*Rep*) gene and the *V1* gene, both of which are positioned adjacent to a conserved intergenic region (IR). For geminiviruses, expression of genes in the v-sense orientation is generally activated during the late stage of infection, under the control of a promoter commonly designated as the late promoter. It has been shown that in bipartite begomoviruses such as tomato golden mosaic virus (TGMV) and ACMV, late promoter activity is generally weaker than early promoters, which drive gene expression in the c-sense orientation (Petty et al., 1988; Zhan et al., 1991; Eagle and Hanley-Bowdoin, 1997). Similar observations have been reported for mungbean yellow mosaic virus (MYMV), where v-sense transcripts from both DNA-A and DNA-B are markedly less abundant during early infection, suggesting that their upstream promoters require activation by the AC2 transcriptional activator. In MYMV DNA-B, AC2 has been shown to trans-activate transcription bidirectionally via a shared cis-regulatory element within the IR. However, the extent of AC2-mediated activation differs between orientations, with the v-sense “late” promoter exhibiting a substantially higher activity than the c-sense “early” promoter (Shivaprasad et al., 2005). Consistent with previous findings, the IR of AYVV-NT, encompassing the region from C1 to V1 ORF (nt 2603–131), exhibited higher basal promoter activity in the c-sense orientation than in the v-sense in *E. coli*, independent of viral transactivators. Interestingly, truncation of nt 53–131 in the IR resulted in a pronounced increase in v-sense promoter activity across all three *E. coli* strains, whereas no significant change in c-sense promoter activity was detected under the same condition (Fig. 5). This observation suggested that the nt 53–131 region may harbor a cis-acting operator element recognized by a host-derived repressor in *E. coli*, which selectively suppresses transcription in the v-sense direction. Removal of this region likely disrupts repressor binding, thereby relieving suppression and enhancing promoter activity. During natural infection in plants, geminiviral genome remain intact without undergoing deletions. Therefore, activation of the v-sense promoter likely depends on the binding of the viral C2/AC2 protein to downstream regulatory elements. Our findings support the hypothesis that C2 protein may activate v-sense gene transcription through both direct DNA binding and interactions with host transcriptional factors, as proposed in previous studies (Shivaprasad et al., 2005; Lacatus and Sunter, 2008; Borah et al., 2016). In our assay system, which lacks C2 expression, promoter de-repression occurred solely via sequence deletion, and notably, the c-sense promoter remained unaffected. Based on these observations, we propose that the AYVV-NT IR contains operator-like elements recognized by host-derived transcriptional repressors in both prokaryotic and eukaryotic systems. Such bidirectional regulatory architecture may ensure the coordinated temporal control of early and late genes expression, while also supporting the cross-kingdom transcriptional compatibility characteristic of geminiviruses.

In this study, we found that the same promoter fragment drove markedly different levels of GFP expression across multiple *E. coli* strains, despite identical culture conditions, suggesting strain-specific differences in transcriptional regulation. However, the different GFP fluorescence might also result from the differences in plasmid copy number, GFP mRNA or protein stabilities in different *E. coli* strains. Accordingly, we have performed qPCR and RT-qPCR (Fig. 3) to estimate transcriptional activities, using *dxs* gene as an internal reference to normalize the variations. Because *dxs* is present as a single-copy gene in *E. coli* genome, it may serve as a reliable internal reference for normalization (Lee et al., 2006).

Prior reports have shown that specific mutations in sigma factors can shift promoter recognition specificity and influence transcription initiation dynamics (Gardella et al., 1989; Zuber et al., 1989; Tomatis et al., 2019). In the current study, c-sense promoter activity was detected exclusively in *E. coli* BL21(DE3), with no detectable expression in DH5α or TOP10. This strain-specific expression pattern suggests that additional regulatory components unique to BL21(DE3) may contribute to promoter activation. These findings highlight a complex interaction between host transcriptional machinery and viral promoter elements, warranting further investigation into host-specific factors that modulate geminiviral promoter activity in prokaryotic systems.

The bidirectional promoter activity of a concise 36-nucleotide fragment (nt 2728–10) within the AYVV-NT IR was demonstrated in this study (Fig. 5). Promoter prediction analysis revealed overlapping −10 elements embedded on opposing strands (Fig. 6), indicative of a compact, bidirectional promoter architecture. The sequence-level overlap of core elements suggests that this fragment may serve as a naturally evolved platform for driving divergent transcription (Fig. 6). This structural arrangement echoes the architecture of the T7 promoter, a minimal 19-bp sequence containing *a* −10 region and defined TSS, which operates independently of a −35 region and is specifically recognized by T7 RNA polymerase in prokaryotic systems. The sufficiency of such streamlined promoters to drive robust transcription has been well documented, particularly in the context of recombinant protein production and large-scale *in vitro* mRNA synthesis (Rong et al., 1998; Komura et al., 2018; Sari et al., 2024). In light of this, the minimal AYVV-NT IR fragment may likewise constitute a compact and bidirectionally active regulatory module that offers functional versatility and potential applicability in synthetic biology and engineered gene expression systems. The activity of AV3 promoter, which is used as the positive control in this study, has been shown to be comparable to that of the strong constitutive *rrnB* P1 promoter of *E. coli* (Csordás-Tóth et al., 1979) in previous study (Wang et al., 2013). Although the activities of the promoter elements in IR of the AYVV-NT are apparently lower than AV3 promoter, these promoter elements identified in this study may still be usable, since we have characterized the important features such as the directionality and associated regulatory elements. The weak promoters might be useful in the following applications such as expression of regulatory proteins (Elowitz and Leibler, 2000), managing metabolic burden (Cardinale and Arkin, 2012), and balancing metabolic pathway (Ajikumar et al., 2010).

The partial overlap of core promoter elements within this bidirectional promoter fragment introduces potential regulatory conflicts in gene expression. Our data consistently show that promoter activity in the v-sense orientation is markedly higher than in the c-sense, suggesting the possibility of simultaneous but asymmetric transcription initiation. This observation raises the possibility of competitive recruitment of RNA polymerase complexes to the shared −10 region. In bacterial systems, transcription initiation depends on the binding of RNAP holoenzyme (Eσ) —comprising the core enzyme and a σ factor—to the −10 element, followed by local DNA melting to form an open complex (Feklistov and Darst, 2011). We propose that the overlapping bidirectional promoter permits dual recognition events, with a preferential affinity for the v-sense configuration, resulting in directional bias in transcriptional output. Several features collectively define this bidirectional promoter region: (1) The 36-nt fragment supports GFP expression in both c-sense and v-sense directions via partially overlapping −10 elements. The pronounced expression bias toward the v-sense direction implies competitive binding of RNAP to the shared site, consistent with its function as an actual bidirectional promoter. (2) Inclusion of an extended fragment (nt 2712–53), which retains predicted −35 elements in both directions, led to a substantial increase in transcriptional activity, reinforcing the presence of functional cis-regulatory elements required for efficient bidirectional initiation. (3) Notably, no apparent AT-tract was identified between the −10 and −35 elements (typically located −17 to −23 bp), indicating that promoter activation likely relies on canonical σ⁷⁰ recognition rather than nonspecific AT-rich DNA interactions (Warman et al., 2020).

While bidirectional promoters are a well-documented feature in prokaryotes, their mechanistic basis differs sharply from those observed in eukaryotes. In bacteria, the symmetry within core promoter sequences—particularly the −10 element—often enables transcription to proceed in both directions from the shared site (Warman et al., 2021; Middlemiss et al., 2023). Such promoter arrangements are frequently associated with horizontally acquired genes, where the AT-rich content promotes the emergence of cryptic −10-like motifs, which can lead to promiscuous and non-specific transcription initiation (Warman et al., 2021; Jin et al., 2017). In eukaryotic systems such as yeast, promoter regions often exhibit intrinsic bidirectionality, producing numerous cryptic unstable transcripts (CUTs) that are rapidly degraded (Neil et al., 2009). Such pervasive transcription events suggest that bidirectional promoters are a widespread yet previously underappreciated regulatory feature in eukaryotic transcription, with the potential to interfere with sense-strand gene expression. Taken together, our findings support the notion that bidirectional promoters are a prevalent and functionally significant feature of transcriptional regulation in prokaryotic systems, particularly in horizontally transferred DNA. These compact promoter elements maximize genomic economy while offering a flexible platform for rapid evolution of gene regulation. Dissecting how these promoters are differentially recognized across host species will be crucial to elucidating how geminiviruses fine-tune their genes expression across both prokaryotic and eukaryotic contexts.

Another noteworthy observation is the identification of promoter activity within the nt 2603–2713 region of the AYVV-NT IR. Promoter prediction analysis revealed distinct −10 and −35 elements in both the c-sense and v-sense orientations, which may be recognizable by bacterial transcriptional machinery (Fig. 6). Results of experimental validation using western blotting and quantitative fluorescence assays showed that the v-sense promoter exhibited a distinct expression profile in *E. coli* BL21(DE3) compared to DH5α and TOP10. Although this promoter-containing region does not overlap with the previously identified bidirectional promoter (nt 2712–53), the two regions together span the broader region of nt 2603–53 originally tested. Comparative analyses of these constructs (Fig. 4) revealed that deletion of one promoter resulted in a marked increase in the activity of the other, in both orientations. This suggests a form of intramolecular interference—possibly driven by competition for σ⁷⁰ or other sigma factors—that limits RNAP access when both promoters are intact. Further analysis of strain-specific expression patterns suggests that the transcriptional machinery in different *E. coli* strains exhibits distinct preferences for v-sense promoters. In DH5α and TOP10, GFP expression predominantly driven by the promoter within the nt 2603–2713 segment, whereas in BL21(DE3), the bidirectional promoter in the nt 2712–53 segment was more actively utilized. However, the corresponding predominant promoter in plants is currently not clearly investigated. This differential usage suggests that variations in transcription factors abundance, structure, or availability—along with other regulatory components—may shape promoter recognition specificity among different strains. These results illustrate the functional diversity of bacterial transcription systems and emphasize the pivotal role of host genetic background in shaping promoter-driven gene expression.

Overall, this study characterized the IR of the AYVV-NT genome through the prokaryotic expression system, revealing two discrete promoter-active regions oriented in both the c- and v-sense directions. Among these, a 36-nt segment was found to harbor a bidirectional promoter capable of driving gene expression from overlapping core motifs in opposite orientations. In addition, we identified putative regulatory elements that may regulate early and late gene expression. By leveraging a prokaryotic GFP reporter system, we demonstrated that these viral promoter elements are transcriptionally active in the absence of eukaryotic host factors or virus-encoded proteins, with notable differences in promoter usage in different *E. coli* strains. Given the highly compact nature of geminiviral genomes, which must encode multiple proteins within limited genomic space, we propose that the evolutionary retention of a bidirectional promoter enhances both the efficiency and flexibility of gene expression. Such promoters, combined with transcription factor binding sites recognizable across biological kingdoms, likely enable geminiviruses to initiate transcription and replication in both prokaryotic (possibly in mitochondria and chloroplasts) and eukaryotic (in nuclei) systems—reflecting a highly adaptable transcriptional regulation strategy at the very early stage of the infections. Furthermore, the ability of these viral promoters to constitutively drive downstream gene expression in prokaryotic hosts without the need for induction highlights their practical potential. These features suggest that such elements could be repurposed as novel vector components for heterologous gene expression in synthetic biology and biotechnology applications.

## Funding

This work was funded by the National Science and Technology Council, Taiwan (NSTC 114-2313-B-005-030-MY3), and the Advanced Plant Biotechnology Center from the Featured Areas Research Center Program within the framework of the Higher Education Sprout Project by the Ministry of Education (MOE).

## CRediT authorship contribution statement

**Hsiao-En Lin:** Writing – original draft, Methodology, Investigation, Formal analysis, Data curation, Conceptualization. **Ying-Wen Huang:** Resources, Methodology. **Yi-Chin Lai:** Validation, Resources. **Na-Sheng Lin:** Writing – review & editing, Conceptualization. **Yau-Heiu Hsu:** Writing – review & editing, Supervision, Funding acquisition, Conceptualization. **Chung-Chi Hu:** Writing – review & editing, Supervision, Funding acquisition, Conceptualization.

## Declaration of competing interest

The authors declare that they have no known competing financial interests or personal relationships that could have appeared to influence the work reported in this paper.

## Data Availability

Data will be made available on request.
